# Single minimum incision endoscopic radical nephrectomy for renal tumors with preoperative virtual navigation using 3D-CT volume-rendering

**DOI:** 10.1186/1471-2490-10-7

**Published:** 2010-04-14

**Authors:** Takao Kamai, Nobutaka Furuya, Tsunehito Kambara, Hideyuki Abe, Mikihiko Honda, Yasukazu Shioyama, Yasushi Kaji, Ken-Ichiro Yoshida

**Affiliations:** 1 Department of Urology, Dokkyo Medical University, Mibu, Tochigi, Japan; 2 Department of Radiology, Dokkyo Medical University, Mibu, Tochigi, Japan

## Abstract

**Background:**

Single minimum incision endoscopic surgery (MIES) involves the use of a flexible high-definition laparoscope to facilitate open surgery. We reviewed our method of radical nephrectomy for renal tumors, which is single MIES combined with preoperative virtual surgery employing three-dimensional CT images reconstructed by the volume rendering method (3D-CT images) in order to safely and appropriately approach the renal hilar vessels. We also assessed the usefulness of 3D-CT images.

**Methods:**

Radical nephrectomy was done by single MIES via the translumbar approach in 80 consecutive patients. We performed the initial 20 MIES nephrectomies without preoperative 3D-CT images and the subsequent 60 MIES nephrectomies with preoperative 3D-CT images for evaluation of the renal hilar vessels and the relation of each tumor to the surrounding structures. On the basis of the 3D information, preoperative virtual surgery was performed with a computer.

**Results:**

Single MIES nephrectomy was successful in all patients. In the 60 patients who underwent 3D-CT, the number of renal arteries and veins corresponded exactly with the preoperative 3D-CT data (100% sensitivity and 100% specificity). These 60 nephrectomies were completed with a shorter operating time and smaller blood loss than the initial 20 nephrectomies.

**Conclusions:**

Single MIES radical nephrectomy combined with 3D-CT and virtual surgery achieved a shorter operating time and less blood loss, possibly due to safer and easier handling of the renal hilar vessels.

## Background

Thanks to various technical and imaging innovations, pure laparoscopic or hand-assisted laparoscopic surgery is now performed worldwide for radical nephrectomy and is considered to be safe and effective, while also improving the quality of life [1,2]. However, laparoscopy still requires three to four incisions, each of which is about 1-2 cm long. Every incision is associated with the risk of bleeding, hernia, and/or internal organ damage, and incrementally worsens the cosmetic outcome [3,4]. Furthermore, several problems remain to be solved with regard to laparoscopy, including the use of CO_2_, the size of the incision for retrieving the resected specimen, the need for trocar ports, and the high cost of equipment. Alternatives to conventional laparoscopy include single-site surgery, which is known as laparo-endoscopic single-site surgery (*LESS*), as well as natural orifice transluminal endoscopic surgery (NOTES). In 1998, Kihara *et al*. reported on minimum incision endoscopic surgery (MIES) performed via a single small incision in Japan, which was an attempt to solve the above-mentioned problems and reduce technical difficulties [5-9]. MIES is performed via a single small incision that just permits extraction of the resected specimen, and is done without gas or trocar ports [5-8]. Kihara *et al*. subsequently reported that radical nephrectomy by single MIES was a safe, reproducible, cost-effective, and minimally invasive treatment option for T1-3aN0 M0 renal tumors [6]. In April 2001, we started to employ single MIES for nephrectomy [9], partial nephrectomy, nephroureterectomy, adrenalectomy, and radical prostatectomy, with two main operators (T.K* and K-I.Y) performing the procedures. In 2006, MIES was recognized as a method of advanced surgery by the Japanese government and it has been covered by the Japanese universal health insurance system since 2008 [5]. Single MIES is based on both standard open surgery and modified hand-assisted laparoscopic surgery [5], so the instruments employed are the same as those used for open surgery or laparoscopic surgery and it only requires a short time to learn the technique [5-7]. Because MIES is performed via a small incision, the surgical field is very limited. Accordingly, complete information about the renal tumor, renal vessels, and adjacent structures needs to be obtained preoperatively so that the surgeon can select the appropriate procedure. Moreover, the renal arteries and veins show anatomical variations that must be clarified before attempting surgical treatment, in order to reduce the risk of unexpected bleeding. It has been reported that three-dimensional (3D) reconstruction of CT images (3D-CT) and/or 3D-CT angiography (CTA) are useful modalities for viewing the renal arteries that are less invasive than conventional angiography [10-12], and can be utilized for navigation when retroperitoneal laparoscopic nephrectomy is performed for renal tumors [13]. Since 2003, we have performed preoperative 3D-CT for evaluation of the renal arteries and veins, as well as the relations between the renal hilar vessels and adjacent structures, in order to improve the outcome of MIES nephrectomy. In the present study, we reviewed the results of MIES radical nephrectomy for renal tumors in 80 consecutive patients treated by one chief operator (T.K*). We assessed the usefulness of 3D-CT images and the method of safely approaching the renal hilum during MIES nephrectomy.

## Methods

### Patients

Eighty Japanese patients aged from 31 to 82 years (mean age: 60.3 years) with cT1-3aN0 M0 renal tumors diagnosed between April 2001 and August 2009 underwent translumbar radical nephrectomy by single MIES before receiving any other therapy. Patients with dorsal tumors located very close to the renal hilar vessels were excluded, because the proximity of such tumors to the renal hilum makes them unsuitable for the translumbar approach. All patients underwent imaging (CT and/or MRI) prior to radical nephrectomy to obtain staging information. The tumor grade and clinical stage were assigned according to the Fuhrman grading system and the TNM classification, respectively [14,15]. Table 1 summarizes the patient demographic data, side and size of tumors on CT, number of renal arteries and veins on CT and at surgery, operating time, and blood loss. The first 20 nephrectomies were performed without 3D-CT data and the subsequent 60 nephrectomies were done after preoperative virtual surgery employing 3D-CT data. Comparison between groups of equal size is preferable for statistical evaluation. Therefore, to assess the influence of preoperative treatment planning with 3D-CT, we divided the subsequent 60 nephrectomy procedures into three chronological groups of 20, giving us a total of four groups of 20 patients. The operating time and blood loss in each group was analyzed in relation to tumor size, side, and location, as well as the skin incision and body mass index (BMI) [16]. This study was conducted in accordance with the Helsinki Declaration. Institutional Review Board approval was obtained for this investigation and each patient signed a consent form approved by the Committee on Human Rights in Research of our institution.

**Table 1:** Data collection from 3D-CT and surgical procedures

	pre-operative axial CT (n = initial 20)	pre-operative 3D-CT (n = subsequent 60)	Surgical procedures (n = 80)
**Patient demographics**			
No. of patients	20	60	80
Age			60.8 (31 - 82)
Sex (male/female)			54/26

**Tumor**			
Tumor side (right/left)			42/38
Tumor size on CT (cm)	4.5 (2 - 12)		

**No. of renal hilar vessels**			
Renal arteries (1/2 or 3)	18/2	53/7	17/3, 53/7
Renal veins (1/2 or 3)	19/1	52/8	17/3, 52/8

**MIES Operation**			
Operative time (min)			131 (77 - 220)
Blood loss (ml)			215 (9 - 1010)

### 3D-CT and preoperative virtual surgery

**Figure 1:**
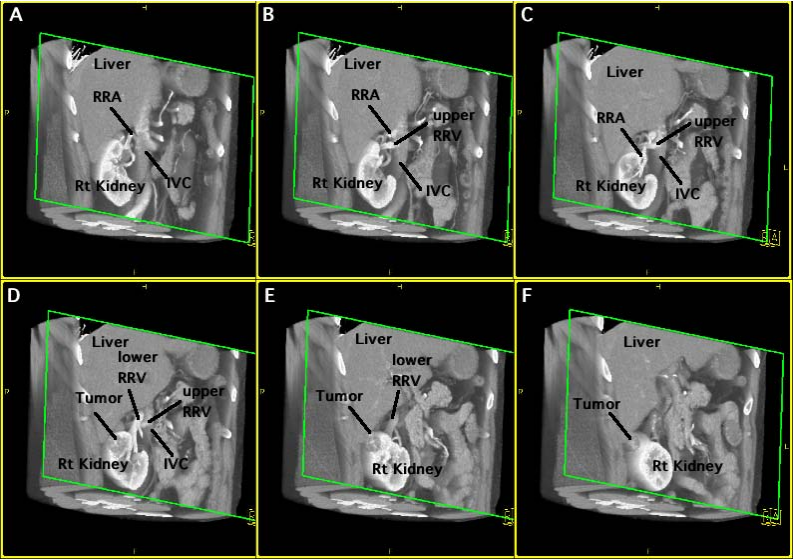
**A right renal tumor and two renal veins**. Oblique view from the ventral to dorsal direction in the arterial phase. The CT scans are arranged from dorsal (A) to ventral (F). A: The right renal artery (RRA) is seen to the right of the inferior vena cava (IVC). B: The upper right renal vein (RRV) can be seen. C: The anterior branches of the RRA and RRV cross each other. D: The lower RRV drains into the IVC closer to the heart than the upper RRV, indicating that these both veins cross over each other. E, F: The tumor is located in the upper pole of the kidney.

**Figure 2:**
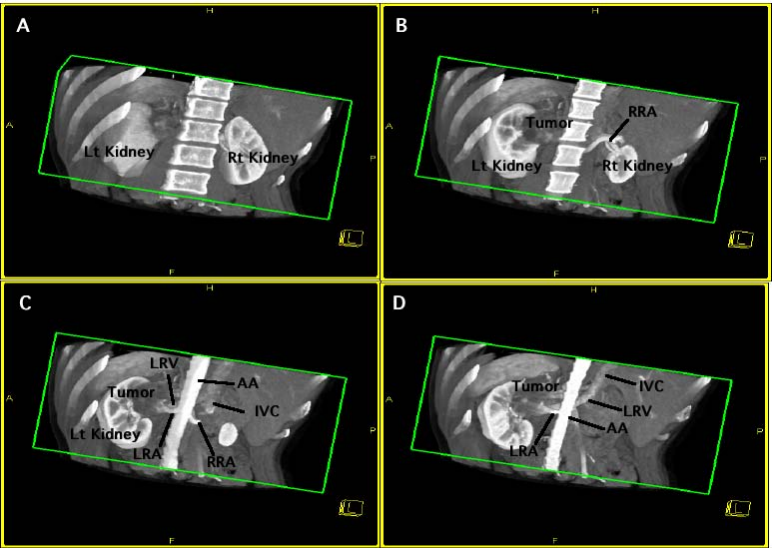
**A left renal tumor adjacent to the renal artery and vein**. Oblique view from the dorsal to ventral direction in the arterial phase. The CT scans are arranged from dorsal (A) to ventral (D). A: The tumor can be seen in the upper pole to middle part of the kidney. B: The tumor is more clearly visualized in the ventral images. The right renal artery (RRA) is also seen. C: The left renal artery (LRA) arises vertically from the abdominal aorta (AA), runs dorsal to the left renal vein (LRV), and drains into the inferior vena cava (IVC), indicating that the LRA and LRV cross each other antero-ventral to the tumor. D: The LRA, LRV, and IVC are seen more clearly in the ventral images.

We usually perform radical nephrectomy via the translumbar approach in patients with relatively small renal tumors [9,17,18]. We did not obtain 3D-CT images in the initial 20 patients, but did so in the subsequent 60 patients (who all had normal renal function and no allergy to contrast medium) to devise an appropriate and safe approach to the renal arteries and veins [9]. All of the axial CT scans were carefully evaluated before 3D reconstruction was performed, and then 3D images were created by software in the CT scanner (Leonardo InSpace, Siemens Healthcare, Forchheim, Germany). The arterial phase was used to depict both the renal arteries and veins, since it is the most sensitive phase for the detection of multiple arteries and veins as well as other abnormalities [19]. Data from multidetector row CT scans were employed to construct the 3D images, after which virtual surgery was performed. Using the 3D images, the location of the kidney in relation to the lower ribs, the iliac crest, and the spine was determined to help the surgeon select the best site for the incision. Possible involvement of surrounding structures by each tumor was also investigated. Images were created that gave an oblique view from the skin incision to the targeted renal artery and vein, in order to allow the surgeon to better understand the anatomical relations between the renal hilar vessels and the surrounding structures. The software (Leonardo InSpace or free software OsiriX) allowed free rotation of the kidney into different positions and facilitated the creation of any desired cut plane, so the relation of the tumor to the renal vessels and adjacent structures could be demonstrated clearly. Virtual surgery was started by making an oblique intercostal incision between the 11 and 12th ribs. After dissecting between the psoas muscle and Gerota's posterior fascia, we approached the kidney anterior to the psoas muscle (Figure. 1, 2). On the right side, we found the posterior surface of the inferior vena cava (IVC) behind the psoas muscle at the level of the lower pole of the kidney. Then we advanced along the IVC toward the liver and identified the right renal artery (RRA). At this level, we also found the right renal vein (RRV) branching from the right side of the IVC. When operating on the left side, we found the left renal artery (LRA) running vertical to the psoas muscle at level of the middle part of the kidney, after which we identified the left renal vein (LRV) behind (ventral to) the LRA. We usually performed the simulated procedure with oblique images from the dorsal to ventral direction as in actual surgery, but we also assessed oblique images from the ventral to dorsal direction in order to better understand the relation between the renal vessels and the tumor or adjacent structures.

### Surgical technique

We performed radical nephrectomy by single MIES via the translumbar approach with the patient in the flank position over the break of the operating table according to Kihara's method [5-7]. The surgical team consisted of the chief operator (T.K*: 19 years of urological experience) and two or three assistants. A flexible high-definition laparoscope (Olympus, Tokyo, Japan) was manipulated by one of the assistants and was moved to the best position for viewing the operating field. The chief operator and first assistant employed a combination of video images and direct vision, while only video images were available for the other assistants.

Based on 3D treatment planning, an oblique intercostal skin incision was made between the 11 and 12th ribs with an average length of about 6 cm (4.5-10), and a wound retractor (2.5-6 cm or 5-9 cm in diameter, Applied Medical, CA) was attached. After the external and internal oblique muscles were split, the transversalis fascia was incised, and dissection was performed between the psoas fascia and Gerota's posterior fascia to approach the kidney anterior to the psoas muscle. Gerota's posterior fascia was bluntly dissected and pushed medial to the psoas muscle, achieving immediate access to the renal arteries and veins.

**Figure 3:**
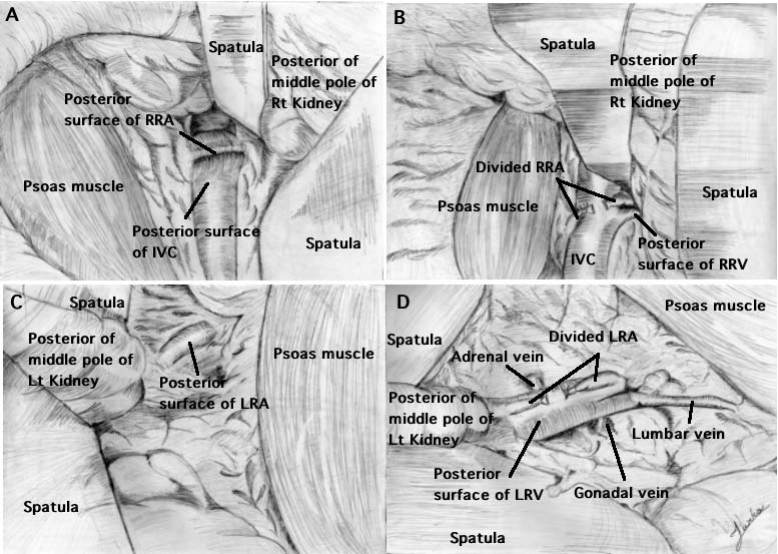
**Sketches of the surgical field viewed through the narrow incision for MIES**. A: Beneath the psoas muscle, the posterior surface of the right renal artery (RRA) is seen running across the posterior surface of the inferior vena cava (IVC) (surrounded by fibrous connective tissue) at the level of the middle part of the right kidney. B: Behind the divided RRA, the posterior surface of the right renal vein (RRV) arises vertically from the right side of the IVC. C: Beneath the psoas muscle, the posterior surface of the left renal artery (LRA) runs vertically in the fibro-fatty connective tissue at the level of the middle part of the left kidney. D: Behind the divided LRA, a complicated venous plexes is found, including the posterior surfaces of the gonadal, adrenal, and left renal veins (LRV), and the anterior surface of the lumbar vein.

On the right side, we first identified the posterior surface of the IVC behind the psoas muscle at the level of the lower pole of the right kidney. After dissecting along the posterior surface of the IVC upward in the direction of the liver, we found fibrous connective tissue overlying the IVC at the level of the middle part of the kidney, under which arterial pulsation could be observed or palpated. This identified the RRA running across the posterior surface of IVC in the surrounding fibrous connective tissue (Figure. 3A). After identifying the posterior surface of the RRA, we dissected the artery from the IVC until it was circumferentially mobilized. Then the RRA was double-ligated and divided. Next, we dissected the right side of the IVC and identified the posterior surface of the RRV, which branches vertically from the IVC behind the divided RRA. After the RRV was freed, it was double-ligated and divided (Figure. 3B).

On the left side, we usually first identified the lumbar vein running vertically toward the psoas muscle at level of the lower to middle part of the kidney. If necessary, this vein was ligated and divided. Near this vein, arterial pulsation could be observed or palpated in the fibro-fatty connective tissue. This identified the posterior surface of the LRA running vertical to the psoas muscle. Since the LRA is usually surrounded by perihilar fibrous connective tissue and lymphatics, we dissected this tissue and removed the lymphatics to identify the posterior surface of the artery (Figure. 3C). After careful circumferential mobilization, ligation and division of the LRA was performed, revealing a complicated venous plexus that contained the LRV and its adrenal, gonadal, and lumbar branches. After the adrenal, gonadal, and lumbar branches were ligated and divided, the main LRV was double-ligated and divided (Figure. 3D). There is one point that requires careful attention. If Gerota's posterior fascia is pushed too far medially off the psoas muscle along with the kidney, it is sometimes hard to find the pulsation of the renal artery because the vessel becomes excessively stretched between the abdominal aorta and renal hilum.

After completing the management of the renal arteries and veins, dissection was performed between the peritoneum and Gerota's anterior fascia to access the renal hilum and the adrenal gland, which was removed together with the kidney when necessary. Then the ureter was ligated and divided, the remaining renal attachments were freed, and the resected kidney was extracted through the incision. Any enlarged lymph nodes were also resected. After placing a drain tube, the incised skin was closed.

### Statistical analysis

Since the data did not show a normal distribution, the results were analyzed statistically by employing the non-parametric Mann-Whitney *U *test for comparisons between two groups and the non-parametric Kruskal-Wallis test to compare four groups. Because Boneferroni's correction is generally employed for multiple comparisons, the Mann-Whitney *U *test was corrected by this method. Spearman's rank correlation coefficient analysis was employed to assess the relations between operating time, blood loss, and tumor size. A probability (P) value of less than 0.05 was considered significant. Analyses were done with commercially available software.

## Results

Nephrectomy was performed successfully by single MIES in all 80 patients. The baseline demographic, clinicopathological, intraoperative, and postoperative data of the subjects are summarized in Tables 2 and 3. Although there were no operative complications, we extended the incision by 1 to 2 cm in four patients (for extraction of large tumors in two and to control hemorrhage from the left renal veins in two). Bleeding was successfully controlled in the two patients with left renal vein hemorrhage and no patient required blood transfusion.

**Table 2:** Backgrounds of the tumors

	1st-20 sets	2nd-20 sets	3rd-20 sets	4th-20 sets
**Side (right/left)**	8/12	9/11	11/9	14/6

**Maximum length on CT (cm)**	4.8 ± 2.6	3.8 ± 1.3	4.7 ± 2.3	5.0 ± 2.3

**Ventral/Dorsal**	15/5	10/10	9/11	12/8

**Upper/Lower**	16/4	10/10	11/9	15/5

**Table 3:** Baseline demographics, intraqoperative and postoperative data

	Our case	Kihara et al, 2004
**Basekine demographics**		
No. of patients	80	80
Age	60.8 (31 - 82)	59.7 (35 - 89)
Sex (male/female)	54/26	58/22
Side (right/left)	42/38	37/43
Tumor size on CT (cm)	4.5 (2 - 12)	4.1 (1 - 9)

**Intraoperative data**		
Incision (cm)	6.2 (5 - 10)	6.6 (4 - 9)
Operative time (min)	131 (77 - 220)	186 (102 - 336)
Blood loss (ml)	215 (9 - 1010)	324 (10 - 2288)
Transfusions	0/80	3/80
Complications	0/80	2/80

**Postoperative data**		
Days to oral feeding	1.4 (1 - 3)	1.4 (1 - 4)
Days to walking	1.5 (1 - 3)	1.4 (1 - 3)

Preoperative 3D volume-rendered image reconstruction was done in 60 patients, revealing that 48 patients had one renal artery and one renal vein, while 12 patients had two or three renal arteries and/or veins. As shown in Table 1, the number of renal arteries and veins detected at operation corresponded exactly with the preoperative 3D-CT findings (100% sensitivity and specificity).

**Figure 4:**
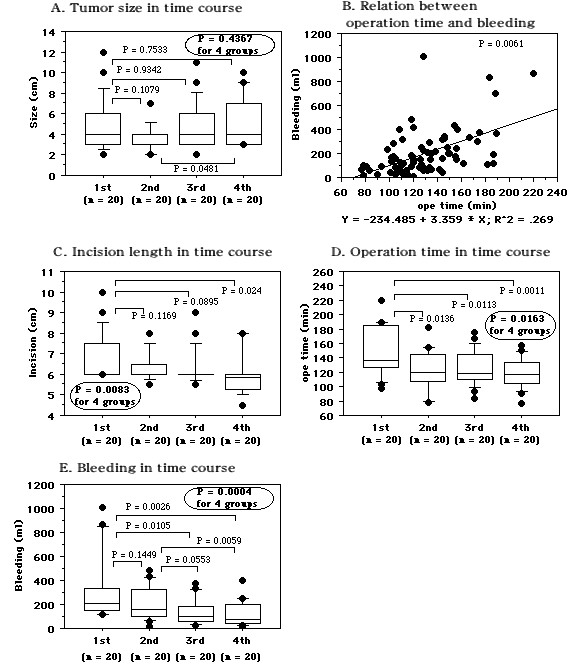
**Comparison of the first to fourth nephrectomy groups (n = 20 each) with regard to tumor size, operating time, blood loss, and incision length**. A: Tumor size. B: Spearman's rank correlation coefficients for the relation between operating time and blood loss. C: Incision length. D: Operating time. E: Blood loss. Median values are shown in the box plots (A, C, D, and E). Bold circled P values were obtained by comparing the four groups with the Kruskal-Wallis test.

**Figure 5:**
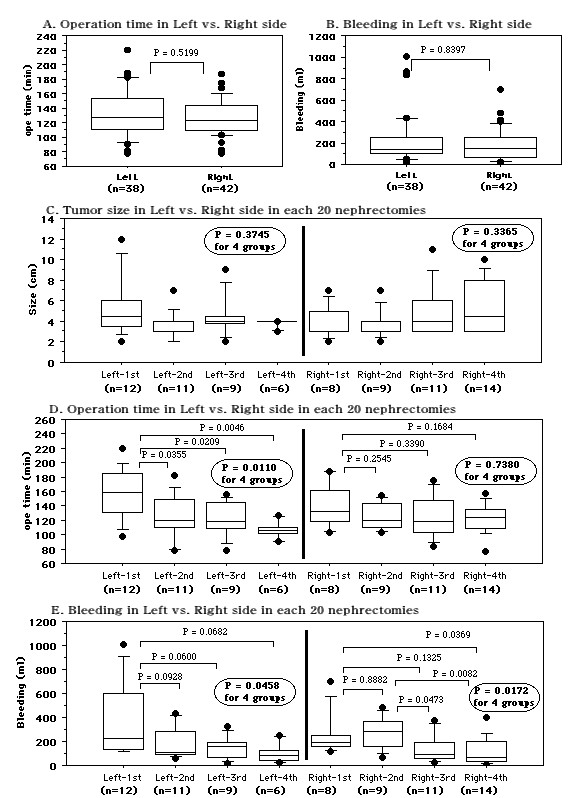
**Comparison of patients from the first to fourth nephrectomy groups with tumors on the left or right side with respect to tumor size, operating time, and blood loss**. A: Operating time. B: Blood loss. C: Tumor size. D: Operating time in each nephrectomy group. E: Blood loss in each nephrectomy group. Median values are shown in the box plots. Bold circled P values were obtained by using the Kruskal-Wallis test to compare the four groups.

The 80 patients were divided into four groups of 20 patients each. The first 20 nephrectomies were performed without 3D-CT data, while the other 60 nephrectomies were done after preoperative virtual surgery was performed with 3D-CT images. There were no differences of tumor size among the four nephrectomy groups (Figure. 4A, Table 2). There was a positive correlation between the operating time and blood loss in all 80 patients (Figure. 4B). The incision was shorter in the second to fourth nephrectomy groups than in the first group (Figure. 4C). Compared with the operating time (mean ± S.D. = 149.7 ± 34.5 min) for the initial 20 nephrectomies performed without virtual surgery, there was a significant decrease in the second (124.8 ± 25.7 min, *P *= 0.0136), third (123.6 ± 27.1 min, *P *= 0.0113), and fourth (118.1 ± 20.3 min, *P *= 0.0011) groups in which nephrectomy was done after preoperative 3D simulation (Figure. 4D). There were no differences of the operating time between the second, third, and fourth groups. Similarly, blood loss in the second (221.6 ± 138.1 ml, *P *= 0.1449), third (144.4 ± 106.7 ml, *P *= 0.0105), and fourth (108.8 ± 103.7 ml, *P *= 0.0026) groups was smaller than in the first group (326.3 ± 282.8 ml, Figure. 4E). There were no differences between nephrectomy on the right and left sides with regard to the operating time (127.0 ± 25.4 vs. 131.3 ± 33.7 min, *P *= 0.5199, Figure. 5A) or blood loss (180.2 ± 147.9 vs. 222.4 ± 228.2 ml, *P *= 0.8397, Figure. 5B). The tumor size was not different among the four groups (Figure. 5C). For left nephrectomy, the operating time was dramatically shorter when the procedure was done after preoperative simulated surgery than when it was done without simulation (the first group), and there was less blood loss in the subsequent three groups than in the first group (Figure. 5D, 5E). For surgery on the right side, blood loss was decreased and the operating time was somewhat shorter when nephrectomy was done after simulated surgery (Figure. 5D, 5E).

**Figure 6:**
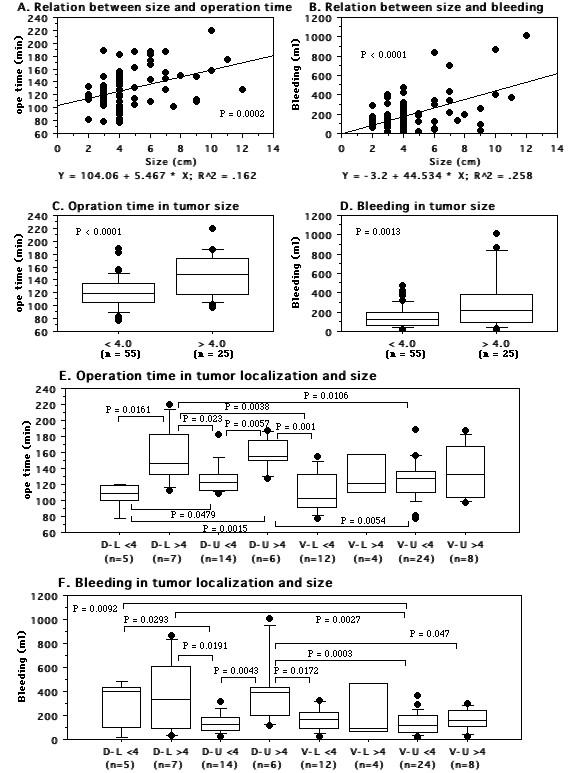
**Comparison of tumor size and tumor location between patients with tumors smaller or larger than 4 cm in diameter**. D-L means dorsal and lower pole tumors. D-U means dorsal and upper pole tumors. V-L means ventral and lower pole tumors. V-U means ventral and upper pole tumors. A: Spearman's rank correlation coefficients for the relation between tumor size and operating time. B: Spearman's rank correlation coefficients for the relation between tumor size and blood loss. C: Operating time. D: Blood loss. E: Operating time in relation to tumor location and size. F: Blood loss in relation to tumor location and size. Median values are shown in the box plots (C to F).

Both the operating time and the blood loss were positively correlated with tumor size (Figure. 6A, 6B). Nephrectomy was more rapid for tumors with a diameter <4 cm (T1a) than for tumors >4 cm in diameter (≥ T1b) (120.7 ± 23.9 vs. 147.4 ± 32.7 min, *P *< 0.0001, Figure. 6C), and was associated with less blood loss (155.1 ± 109.9 vs. 299.6 ± 277.5 ml, *P *= 0.0013, Figure. 6D). We also analyzed the influence of tumor size and location on the operating time and blood loss. Tumors that were dorsal, at the lower pole, and/or large required a longer operating time and were associated with greater blood loss (Figure. 6E, 6F).

**Figure 7:**
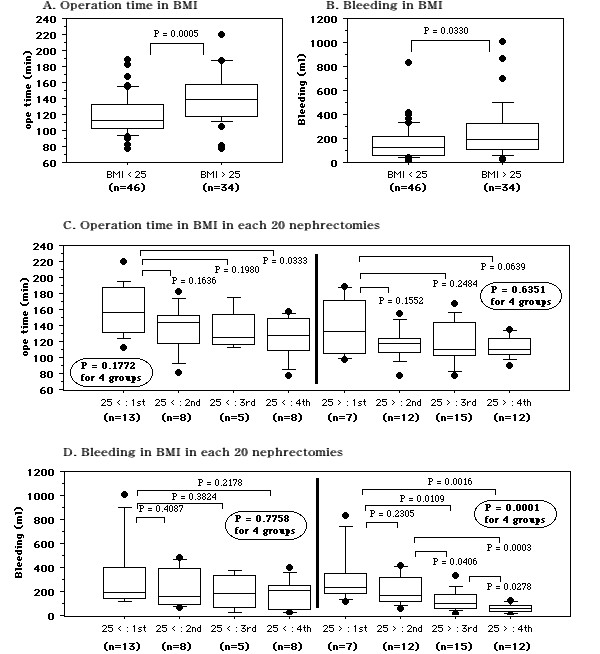
**Comparison of patients with a BMI < 25 or >25 kg/m^2^**. A: Operating time. B: Blood loss. C: Operating time for the first to fourth nephrectomy groups. D: Blood loss of the first to fourth nephrectomy groups. Median values are shown in the box plots. Bold circled P values were obtained by using the Kruskal-Wallis test to compare the four groups.

With regard to BMI, there was a difference of the operating time between patients with a BMI < 25 (n = 46) and those with a BMI > 25 (n = 34) (119.8 ± 24.7 vs. 141.5 ± 31.2 min, *P *= 0.0005, Figure. 7A). There was also a difference of blood loss between patients with a BMI < 25 or >25 (162.8.2 ± 147.9 vs. 251.0 ± 228.5 ml, *P *= 0.0330, Figure. 7B). The operating time tended to get shorter across the four nephrectomy groups in patients with a BMI > 25, while blood loss was smaller in the subsequent groups than the initial group among patients with a BMI < 25 (Figure. 7C, 7D).

## Discussion

Since we were familiar with the anatomical frame, landmarks, and operative technique of radical nephrectomy for cT1-3aN0 M0 renal tumors via the translumbar approach, we also employed this approach for single MIES nephrectomy [9]. Our results demonstrate that radical nephrectomy can be safely performed as a single MIES procedure. As can be seen in Table 3, the background factors, operating time, blood loss, and postoperative recovery time of our series were compatible with previous results reported by Kihara *et al*. [6]. Therefore, as Kihara *et al*. also concluded, radical nephrectomy by single MIES is safe, reliable, and minimally invasive.

The extraperitoneal subcostal translumbar approach avoids the risk of peritoneal contamination and also results in earlier resumption of normal bowel function following surgery. We used images created by 3D-CT to display the location of the kidney in relation to the lower rib cage, iliac crest, and spine, thereby helping the surgeon to accurately plan the initial incision. The position of the kidney and the location and size of the tumor(s) determined the length of the incision.

Preoperative 3D imaging of the renal arteries and veins provides useful information for laparoscopic nephrectomy [13]. Single MIES is performed via a single small incision, detailed anatomical information is required in order to approach the renal artery and vein safely as the operation progresses step-by-step with manipulation of the endoscope and instruments through the narrow single incision. Retrospective analysis of the initial 20 MIES nephrectomies suggested several issues. First, it is very important to define the location and the number of renal arteries and veins, as well as their relations to gain structures, before performing MIES nephrectomy. Second, it is necessary to gain sufficient knowledge in order to accurately approach and locate the renal vessels during MIES nephrectomy, because the renal artery must be ligated and divided before the renal vein. Therefore, it is important to approach the renal artery in a safe and appropriate manner. Third, there are reported to be some anatomical differences of these vessels between the right and left sides (Figure. 3).

In order to overcome these problems, we performed preoperative 3D-CT. We used the volume rendering method for reconstruction of 3D images because it retains all data by summing the contributions from each voxel along a line set at any viewing angle through a stack of axial images. After 3D images have been created, two-dimensional images can also be obtained. That is, the 3D-CT images can be employed to view the kidney in different positions and 2-dimensional images can be created in any desired plane, allowing clear demonstration of the relation between the tumor and the renal vessels or adjacent structures. If perirenal collateral veins are detected in a patient by 3D-CT, we must pay careful attention when dissecting the kidney from the surrounding fibrous connective tissue. Marukawa *et al*. reported that 3D imaging achieved almost perfect detection of renal arteries and veins, and that 3D simulation of retroperitoneal laparoscopic nephrectomy could help surgeons to avoid various operative risks and possible complications [13]. In our study, the number of renal arteries and veins corresponded completely with the preoperative 3D-CT data (100% sensitivity and 100% specificity). With preoperative virtual surgery, we cannot actually perform dissection between the psoas fascia and Gerota's posterior fascia to approach the renal hilar vessels, but performing the virtual procedure is likely to provide more information than that gained from careful study of standard axial CT scans.

Comparison between our first 20 nephrectomy procedures and the next 20 revealed a shorter operating time and smaller blood loss in the latter group, while the third and fourth nephrectomy groups also had a shorter operating time and smaller blood loss than the first group (Figure. 4). Irrespective of the lack of a difference in tumor size or side among patients from the first to fourth groups undergoing right nephrectomy, blood loss was markedly decreased and the operating time was shorter in patients from the second to fourth nephrectomy groups with preoperative virtual surgery than in those from the first group without it (Figure. 5). Also, the operating time was dramatically shorter and blood loss tended to be smaller in second to fourth groups than the first group of patients undergoing left nephrectomy (Figure. 5). These findings suggest that both the approach and the time required to manage the renal hilar vessels differ between the right and left sides. On the right side, the renal artery is easily located on the posterior surface of the IVC at the level of the middle part of the kidney and handling the renal vein is relatively simple, while it takes more time to find the left renal artery and to manage the left renal vein and its branches. Based on data from preoperative 3D simulation, MIES nephrectomy itself, and re-evaluation of our surgical technique by reviewing operative videos for the subsequent 60 nephrectomies, we have developed a successful method of approaching the kidney and handling the hilar vessels. As described in Methods, our surgical approach to the hilar vessels was improved by reviewing the 3D-CT information.

Resection of dorsal, lower pole and/or large tumors took longer and was associated with more blood loss (Figure. 6). In addition to a shorter operating time and smaller blood loss, our results indicated that the incision was smaller in the second to fourth nephrectomy groups than in the first group despite a similar tumor size, and there were no serious complications (Figure. 4C). In particular, for patients with T1a renal tumors 2-3 cm in diameter that were not protruding outside the kidney, we could safely perform MIES nephrectomy via a 4.5-5 cm incision. Furthermore, in comparison to the initial 20 nephrectomies without preoperative simulated surgery, the time to reach renal artery was shorter for the subsequent 60 nephrectomies with simulated surgery (15.8 ± 12.7 vs. 24.1 ± 18.6 min, *P *= 0.0711, data not shown), indicating that we found the vessels at the expected location. Therefore, the use of 3D-CT data not only improves the surgical incision and the approach to the renal hilar vessels, but may also decrease operative complications. The body habitus of the patient is well known to significantly influence the operating times. In the present study, patients with a BMI > 25 had a longer operating time and more bleeding than those with a BMI < 25. Also, the operating time was shorter for patients with a BMI > 25 and bleeding was significantly less for those with a BMI < 25 when the subsequent 60 nephrectomies were compared to the initial 20 nephrectomies (Figure. 7). These results may reflect both the feedback effect and the learning curve related to accumulation of experience with virtual operations and actual MIES nephrectomy, indicating that virtual surgery based on 3D-CT data may be useful for identifying the renal hilar vessels and their relations to adjacent structures, allowing MIES nephrectomy to be performed more safely. However, a randomized trial comparing the outcome for patients with or without preoperative virtual surgery should be performed in order to confirm that simulation employing 3D-CT images is useful.

Single MIES is based on standard open surgery, but we use a flexible high-definition laparoscope for easy identification of tissue planes and more precise dissection with minimal trauma. Many of the longer instruments used in open surgery can be inserted into the narrow incision and employed for single MIES, so it has a lower cost than conventional laparoscopic surgery (30-40% lower). Moreover, the assistants are now performing single MIES as chief operators at our hospital. Because of their experience with the surgical technique, including direct vision and viewing video images as assistants during single MIES procedures, they had a relatively short learning period. Another advantage of single MIES is that the incision can be extended quickly if required.

In patients with a single, small (<4 cm), and localized renal cell carcinoma, nephron-sparing surgery has become more common due to advances in renal imaging, improved surgical techniques, an increase of incidentally discovered low-stage renal cell carcinomas, and good tumor control and potentially better overall survival have been reported in patients undergoing this procedure [20]. Therefore, radical nephrectomy is no longer the standard type of surgery for such tumors and may even be detrimental [20]. It has been reported that 3D-CT provides superior images of the renal vessels and collecting system, and thus is useful for nephron-sparing surgery [10,11,21]. The technique of performing MIES nephrectomy after virtual surgery based on 3D-CT with reconstruction of images by the volume rendering method can also be used for MIES nephron-sparing surgery and MIES adrenalectomy. At present, we perform all of these types MIES after preoperative virtual surgery. MIES nephron-sparing surgery has been increasing every year. We performed this nephron-sparing procedure via a 3-4 cm incision for the above-mentioned renal tumors and have had no complications or local recurrence (data not shown).

Since we have no experience of *LESS *or NOTES, we could not determine whether those procedures or single MIES were superior or not. However, any of these new single-site laparo-endoscopic procedures may be a potential alternative to conventional open or laparoscopic surgery.

## Conclusion

Single MIES radical nephrectomy can be performed more safely by utilizing 3D-CT images for virtual surgery, resulting in a shorter operating time and less blood loss.

## Competing interests

The authors declare that they have no competing interests.

## Authors' contributions

TK* was the chief operator, and initiated the study, participated in its design and coordination, carried out the study, performed the statistical analysis, and drafted the manuscript. NF, TK, HA, and MH were the assistants. TK*, YS, and YK constructed 3D volume-rendered images. KIY participated in the design of the study and helped to draft the manuscript. All authors read and approved the final manuscript.

## Pre-publication history

The pre-publication history for this paper can be accessed here:

http://www.biomedcentral.com/1471-2490/10/7/prepub

## References

[B1] Dunn MD Portis AJ Shalhav AL Elbahnasy AM Heidorn C McDougall EM Clayman RV Laparoscopic versus open radical nephrectomy: a 9-year experience J Urol 2000 164 1153 1159 10.1016/S0022-5347(05)67131-5 10992356

[B2] Rassweiler J Frede T Henkel TO Stock C Alken P Nephrectomy: a comparative study between the transperitoneal and retroperitoneal laparoscopic versus the open approach Eur Urol 1998 33 489 496 10.1159/000019640 9643669

[B3] Raman JD Cadeddu JA Rao P Rane A Single-incision laparoscopic surgery: initial urological experience and comparison with natural-orifice transluminal endoscopic surgery BJU Int 2008 101 1493 1496 10.1111/j.1464-410X.2008.07586.x 18325059

[B4] Kommu S Kaouk JH Rane A Laparo-endoscopic single-site surgery; preliminary advances in renal surgery BJU Int 2008 103 1034 1037 10.1111/j.1464-410X.2008.08282.x 19245444

[B5] Kihara K Kawakami S Fujii Y Masuda H Koga F Gasless single port access endoscopic surgery in urology: Minimum incision endoscopic surgery, MIES Int J Urol 2009 16 791 800 10.1111/j.1442-2042.2009.02366.x 19694839

[B6] Kihara K Kageyama Y Yano M Kobayashi T Kawakami S Fujii Y Masuda H Hyochi N Portless endoscopic radical nephrectomy via a single minimum incision in 80 patients Int J Urol 2004 11 714 720 10.1111/j.1442-2042.2004.00895.x 15379934

[B7] Kageyama Y Kihara K Ishizaka K Okuno T Hayashi T Kawakami S Masuda H Suzuki M Hyochi N Arai G Endoscopic minilaparotomy radical nephrectomy for chronic dialysis patients Int J Urol 2002 9 73 76 10.1046/j.1442-2042.2002.00422.x 12028294

[B8] Kageyama Y Kihara K Kobayashi T Kawakami S Fujii Y Masuda H Yano M Hyochi N Portless endoscopic adrenalectomy via a single minimum incision using a retroperitoneal approach: Experience with initial 30 cases Int J Urol 2004 11 693 699 10.1111/j.1442-2042.2004.00897.x 15379930

[B9] Kamai T Yoshida K Portless endoscopic radical nephrectomy Urology View 2006 4 66 72

[B10] Chernoff DM Silverman SG Kikinis R Adams DF Seltzer SE Richie JP Loughlin KR Three dimensional omaging and display of renal tumors using spiral CT; a potential aid to partial nephrectomy Urololgy 1994 43 125 129 10.1016/S0090-4295(94)80285-8 8284875

[B11] Coll DM Uzzo RG Herts BR Davros WJ Wirth SL Novick AC 3-Dimensional volume rendered computerized tomography for preoperative evaluation and intraoperative treatment of patients undergoing nephron sparing surgery J Urol 1999 161 1097 1102 10.1016/S0022-5347(01)61599-4 10081846

[B12] Coll DM Herts BR Davros WJ Uzzo RG Novick AC Preoperative use of 3D volume rendering to demonstrate renal tumors and renal anatomy Radiographics 2000 20 431 438 10715341 10.1148/radiographics.20.2.g00mc16431

[B13] Marukawa K Horiguchi J Shigeta M Nakamoto T Usui T Ito K Three-dimensional navigator for retroperitoneal laparoscopic nephrectomy using multidetector row computerized tomography J Urol 2002 168 1933 1936 10.1016/S0022-5347(05)64266-8 12394679

[B14] Fuhrman SA Lasky LC Lmas C Prognostic significance of morphologic parameters in renal cell carcinoma Am J Surg Pathol 1982 6 655 663 7180965 10.1097/00000478-198210000-00007

[B15] Sobin LH Wittekind CH editors International union against cancer. UICC TNM classification of malignant tumors 2002 6 New York, Wiley-Liss

[B16] WHO Obesity: preventing and managing the global epidemic. Report of a WHO Consultation. WHO Technical Report Series 894 2000 Geneva: World Health Organization 11234459

[B17] Fukui I Ohashi H Sumi S Satake I Kihara K Takeuchi S Oshima H Translumbar radical nephrectomy of renal cell carcinoma Jpn J Urol 1989 80 1207 1214 10.5980/jpnjurol1989.80.1207 2585920

[B18] Kageyama Y Fukui I Goto S Kitahara S Kamai T Suzuki T Oshima H Treatment results of radical nephrectomy for relatively confined small renal cell carcinoma: translumbar versus transabdominal approach Jpn J Urol 1994 85 599 603 10.5980/jpnjurol1989.85.599 8189657

[B19] Herts BR Coll DM Lieber ML Streem SB Novick AC Triphasic helical CT of the kidneys: contribution of vascular phase scanning in patients before urologic surgery Am J Roentgenol 1999 173 1273 1277 10.2214/ajr.173.5.10541104 10541104

[B20] Novick AC Wein AJ, Kavoussi LR, Novick AC, Partin AW, Peters CA Open surgery of the kidney Campbell-Walsh Urology 2007 9 Philadelphia: Saunders Elsevier 1686 1758

[B21] Ueda T Tobe T Yamanoto S Motoori K Murakami Y Igarashi T Ito H Selective intra-arterial 3-dimensional computed tomography angiography for preoperative evaluation of nephron-sparing surgery J Comput Assist Tomogr 2004 28 496 504 10.1097/00004728-200407000-00010 15232381

